# Risk factors influencing cerebral venous infarction after meningioma resection

**DOI:** 10.1186/s12883-022-02783-2

**Published:** 2022-07-13

**Authors:** Qing Cai, Shoujie Wang, Min Zheng, Xuejiao Wang, Rong Liu, Liqin Liu, Huaizhou Qin, Dayun Feng

**Affiliations:** Department of Neurosurgery, Tangdu Hospital, Air Force Medical University, Xi’an, Shaanxi People’s Republic of China

**Keywords:** Cerebral venous infarction, Risk factors, Meningioma, Prognosis

## Abstract

**Background:**

Cerebral venous infarction (CVI) is a serious complication after meningioma resection. The risk factors of postoperative cerebral venous infarction after surgical resection of meningioma can be determined through large samples and this study can add evidence to the literature.

**Methods:**

The clinical and imaging data of 1127 patients with intracranial meningiomas who underwent resection in our hospital were retrospectively collected and analyzed. CVI was evaluated by postoperative imaging and clinical manifestations. Univariate and multivariate analyses were performed to identify risk factors associated with CVI.

**Results:**

Overall, 4.7% (53/1127) of patients experienced CVI after meningioma resection. Multivariate analysis revealed superficial meningioma, moderate to severe peritumoral edema, peritumoral critical vein and WHO grade II-III as independent predictors of a postoperative CVI. After timely intervention, the symptoms were clearly alleviated in one month, and the prognosis was good, but injury to key veins could cause irreversible neurological disorders.

**Conclusions:**

Intraoperative protection of veins is the primary way to prevent CVI. The present study identified several significant and independent risk factors for postoperative venous infarction, thereby enabling the identification of high-risk patients who require special attention during clinical and surgical management.

## Introduction

Cerebral venous infarction (CVI) is considered to be the most serious complication after meningioma resection [1]. This complication can lead to hemorrhage, epilepsy, limb dysfunction, and even life-threatening conditions. However, due to the many anastomoses and a lack of venous valves [2], its incidence is not high. Even if there is an infarction, there are usually no obvious clinical symptoms, and only a small number of patients have serious symptoms; therefore, it has received little attention.

However, with the increased demand for neurosurgeons to improve their total removal rate of complex skull base meningiomas and parasagittal/falx meningiomas with serious sinus invasion and a lack of understanding of their veins, there will be purposeful or accidental damage to the related peritumoral veins during the tumor resection, which will significantly increase the incidence of venous injury. According to the literature, the incidence of venous injury during intracranial tumor resection is 2.6% to 30%, and the incidence of CVI after injury is 0.15% to 13% [3]. In addition, it has been reported that the incidence of CVI after meningioma resection is 2.0–3.3%, and the infarction rate of superficial meningioma is as high as 5.5%, and these infarctions can cause irreversible neurological damage [4].

Therefore, we should consider the possibility of a serious CVI occurring during meningioma resection. Understanding the risk factors for CVI and a comprehensive evaluation can effectively reduce the incidence of infarctions. In this study, 53 patients with CVI after meningioma resection were analyzed retrospectively. The risk factors related to cerebral infarction were discussed, and corresponding surgical strategies were proposed to reduce the occurrence of CVI.

## Methods

### Patients and data collection

After the approval of the hospital ethics committee and the informed consent of the patients or their families, the clinical and imaging data of 1127 patients with intracranial meningiomas who underwent resection in the Department of Neurosurgery, Tangdu Hospital, Air Force Medical University from March 2011 to July 2020 were retrospectively collected and analyzed. All operations were performed by experienced senior doctors. Age, sex, tumor size, tumor location, peritumoral edema, peritumoral vein, tumor resection degree and pathological grade were included. The size of the tumor was taken as its largest diameter on MRI. The location of the tumors was divided into superficial meningiomas: convexity, parasagittal and falx meningiomas; deep meningiomas: skull base and other meningiomas. Peritumoral edema was divided into two groups: no-mild and moderate-severe. EI (edema index) = edema plus tumor volume/tumor volume (EI = 1, no edema; EI = 1–1.5, mild edema; EI = 1.5–3, moderate edema; EI > 3, severe edema). Peritumoral veins were divided into key veins (Labbe, Trolard, Rolando, etc.) and collateral anastomotic veins. The degree of tumor resection was evaluated by intraoperative and postoperative enhanced MRI. Gross total resection (GTR) was defined as Simpson grades I–III, while subtotal resection (STR) was defined as grades IV–V. Histopathological grading was performed according to the 2016 WHO criteria. The patients were followed up for an average of 6 months. The prognosis of the patient after a venous infarction was evaluated by the GOS score.

CVI (postoperative CT scan) was defined as a new or a larger low-density area around the tumor resection or hemorrhage around the low-density area in the brain parenchyma after the operation. CVI included symptomatic and asymptomatic. Symptomatic CVI was the occurrence of neurological dysfunction, epilepsy and a disturbance of consciousness that matched the venous infarction area. The patient had no clinical manifestations, but the imaging considered that CVI was asymptomatic venous cerebral infarction. In this study, we focused on additional medical or surgical treatment for patients with symptomatic CVI.

### Histopathology and image capture

The tumor specimens were histopathologically examined and analyzed. Hematoxylin-eosin (HE) staining was performed on the 5μm section of the tumor center. Images are observed and captured using equipments including microscopes (LEICA DM 2500), objective lenses (LEICA 10 × /0.25), camera (LEICA DFC310 FX), detector (LEICA CMS GmbH D-35578 Wetzlar) and software (LEICA Application Suite, version 4.0.0). The resolution was measured at 300 dpi with automatic exposure and white balance.

### Statistical analysis

Categorical variables were expressed as numbers (percentages), and the difference was evaluated by the chi-squared test or Fisher’s exact test, as appropriate. To detect independent risk factors associated with the incidence of CVI, univariate regression analysis was adopted. Risk factors with *p* < 0.05 in univariate regression analysis were selected for further multivariate regression analysis. Odds ratios (ORs) along with 95% confidence intervals (CIs) were calculated. All statistical analyses were conducted by using R software version 4.0 (R Core Team, R Foundation for Statistical Computing, Vienna, Austria. http://www.R-project.org/).

## Results

### Incidence and characteristics of postoperative venous infarction

Postoperative venous infarction occurred in 53 of 1127 patients (4.7%) who had undergone surgical resection for meningioma. There were 37 of symptomatic infarction and 16 of asymptomatic infarction. 35 superficial meningiomas (12 convexity, 15 parasagittal and 8 falx) and 18 deep meningiomas (14 skull base and 4 lateral ventricle). Venous infarction was most commonly associated with falx meningioma (10.7%, 8 of 75), followed by parasagittal (6.8%, 15 of 220), convexity (3.5%, 12 of 335), skull base and lateral ventricle (3.6%, 18 of 497). Among 37(3.3%) of symptomatic infarction, 28 superficial meningiomas (8 convexity, 12 parasagittal and 8 falx) and 9 deep meningiomas (7 skull base and 2 lateral ventricle). Venous infarction was most commonly associated with falx meningioma (10.7%, 8 of 75), followed by parasagittal (5.4%, 12 of 220), convexity (2.4%, 8 of 335), skull base and lateral ventricle (1.8%, 9 of 497) (Tables 1 and 2).

### Risk factors related to symptomatic venous infarction

#### Univariate analysis

Patients suffering from superficial meningioma significantly more often exhibited postoperative venous infarction than those with deep meningiomas (71.7% *vs*. 28.3%, *p* = 0.028, OR 2.03, 95% CI 1.12–3.85). Moderate to severe peritumoral edema was significantly more often associated with venous infarction than none to mild peritumoral edema (56.6% *vs*. 43.4%, *p* = 0.001, OR 2.70, 95% CI 1.55–4.78). Patients suffering from peritumoral critical veins significantly more often exhibited postoperative venous infarction than did those with peritumoral collateral veins (67.9% *vs.* 32.1%, *p* < 0.001, OR 3.24, 95% CI 1.82–6.00). Patients with WHO grade II–III meningioma significantly more often suffered from venous infarction than did those with WHO grade I meningiomas (18.9% *vs.* 81.1%, *p* = 0.040, OR 2.29, 95% CI 1.515–3.613) (Table 2).

**Table 1:** Patient and tumor characteristics

Variable	Value
No. of patients	1127
Sex	
Female	724 (64.2%)
Male	403 (35.8%)
Age (year)	
< 60	625 (55.5%)
≥ 60	502 (44.5%)
Tumour size (mm)	
< 40	471 (41.8%)
≥ 40	656 (58.2%)
Tumor localization	
Superficial	630 (55.9%)
Deep	497 (44.1%)
Peritumoural edema	
None to mild	748 (66.4%)
Moderate to severe	379 (33.6%)
Peritumoural vein	
Critical vein	459 (40.7%)
Collateral vein	668 (59.3%)
Extent of resection	
Gross total	934 (82.9%)
Subtotal	193 (17.1%)
Histological grade	
WHO I	1017 (90%)
WHO II–III	110 (10%)

**Table 2:** Risk factors related to the venous infarction after meningioma resection

Risk factor	Venous infarction		Univariable analysis		Multivariable analysis	
	(-)	(+)	OR (95%CI)	*p* Value	OR (95%CI)	*P* value
Sex						
Female	691 (64.3%)	33 (62.3%)				
Male	383 (35.7%)	20 (37.7%)	1.10 [0.61–1.93]	0.872		
Age (years)						
< 60	599 (55.8%)	26 (49.1%)				
≥ 60	475 (44.2%)	27 (50.9%)	1.31 [0.75–2.29]	0.413		
Tumour size (mm)						
< 40	446 (41.5%)	25 (47.2%)				
≥ 40	628 (58.5%)	28 (52.8%)	0.80 [0.46–1.39]	0.503		
Tumor localization						
Deep	479 (44.6%)	15 (28.3%)				
Superficial	595 (55.4%)	38 (71.7%)	2.03 [1.12–3.85]	0.028	1.88 [1.03–3.61]	0.04
Peritumoural edema						
None-Mild	725 (67.5%)	23 (43.4%)				
Moderate-Severe	349 (32.5%)	30 (56.6%)	2.70 [1.55–4.78]	0.001	2.73 [1.54–4.90]	< 0.01
Peritumoural vein						
Collateral	651 (60.6%)	17 (32.1%)				
Critial	423 (39.4%)	36 (67.9%)	3.24 [1.82–6.00]	< 0.001	3.27 [1.83–6.08]	< 0.01
Extent of resection						
Gross total	889 (82.8%)	45 (84.9%)				
Subtotal	185 (17.2%)	8 (15.1%)	0.87 [0.37–1.78]	0.830		
Histological grade						
WHO I	974 (90.7%)	43 (81.1%)				
WHO II-III	100 (9.31%)	10 (18.9%)	2.29 [1.05–4.53]	0.040	2.47 [1.11–5.07]	0.02

#### Multivariate analysis

We performed a multivariate logistic regression analysis to identify potential predictors of postoperative venous infarction in meningioma patients. The presence of superficial meningioma (*p* = 0.04, OR 1.88, 95% CI 1.03–3.61), moderate to severe peritumoral edema (*p* < 0.01, OR 2.73, 95% CI 1.54–4.90) and peritumoral critical vein (*p* < 0.01, OR 3.27, 95% CI 1.83–6.08), WHO grade II-III (*p* = 0.02, OR 2.47, 95% CI 1.11–5.07) could be identified as the only independent and significant predictors of postoperative venous infarction (Table 2).

### Prognosis of symptomatic venous infarction

The main manifestations after infarction were a deterioration of consciousness after hemorrhage (6 cases), headache (10 cases), limb dysfunction (14 cases), epilepsy (7 cases), or aggravation of the original symptoms. The disturbance of consciousness (6 cases) mostly occurred 1–3 days after the operation and showed progressive worsening. 37 cases of symptomatic cerebral venous infarction were divided into mild (20 cases, score of 13–15), moderate (10 cases, score of 9–12) and 7 cases (severe, score of 3–8). 16 cases were asymptomatic (mild, score of 15). The hematoma was removed in emergency situations, and the bone flap was removed when necessary. Other symptoms showed chronic aggravation over 3–7 days and were gradually relieved over 8–14 days. The symptoms were relieved most obviously within 1 month after the operation, but no obvious further improvement was found after 3 months. The overall prognosis was good. After an average follow-up of 6 months, there was 1 case of severe disability (muscle strength grade 0 of one limb, loss of deep and shallow sensation, injury of the Rolando vein), 1 case of vegetative survival (injury of the Labbe vein), and 1 case of death (death due to severe intracranial infection after cerebral venous infarction) (Table 3).

**Table 3:** Prognosis of symptomatic venous infarction after meningioma surgery

GOS score	7 Day (Post-op)	1 Month (Post-op)	3 Month (Post-op)	6 Month (Post-op)
5 (good recovery)	14 (37.9%)	26 (70.3%)	28 (77.8%)	29 (80.5%)
4 (moderate disability)	18 (48.6%)	8 (21.6%)	6 (16.6%)	5 (13.9%)
3 (severe disability)	5 (13.5%)	2 (5.4%)	1 (2.8%)	1 (2.8%)
2 (vegetative state)	0	-	1 (2.8%)	1 (2.8%)
1 (dead)	0	1 (2.7%)	-	-

### Illustrative cases

**Fig. 1:**
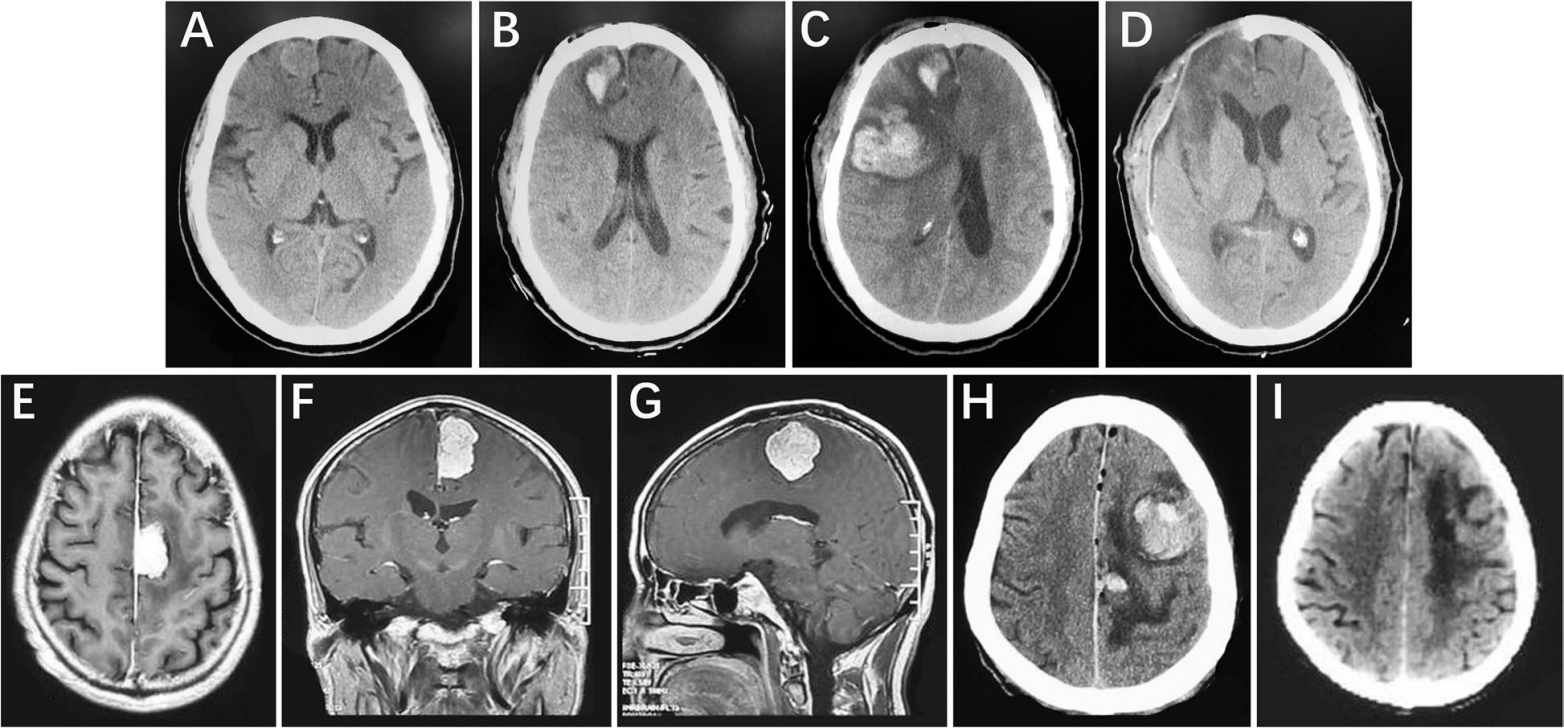
**A** A right frontal parasagittal meningioma, middle front vein injury during operation. **B** A small amount of hematoma in the operative area one day after operation. **C **Massive hemorrhage around the operative area and disturbance of consciousness two day after operation. **D** Cleared hematoma and removed the bone flap, GOS was 5 score one month after operation. **E**-**G** A left parietal falx meningioma, post front vein injury during operation. **H** Bleeding around the operative area one day after operation, right side muscle strength level 2. **I** Cleared hematoma GOS was 5 score one month after operation

Two cases of meningioma with risk factors (critical vein and superficial) caused hemorrhage after venous infarction by damaging critical vein (Fig. 1).

**Fig. 2:**
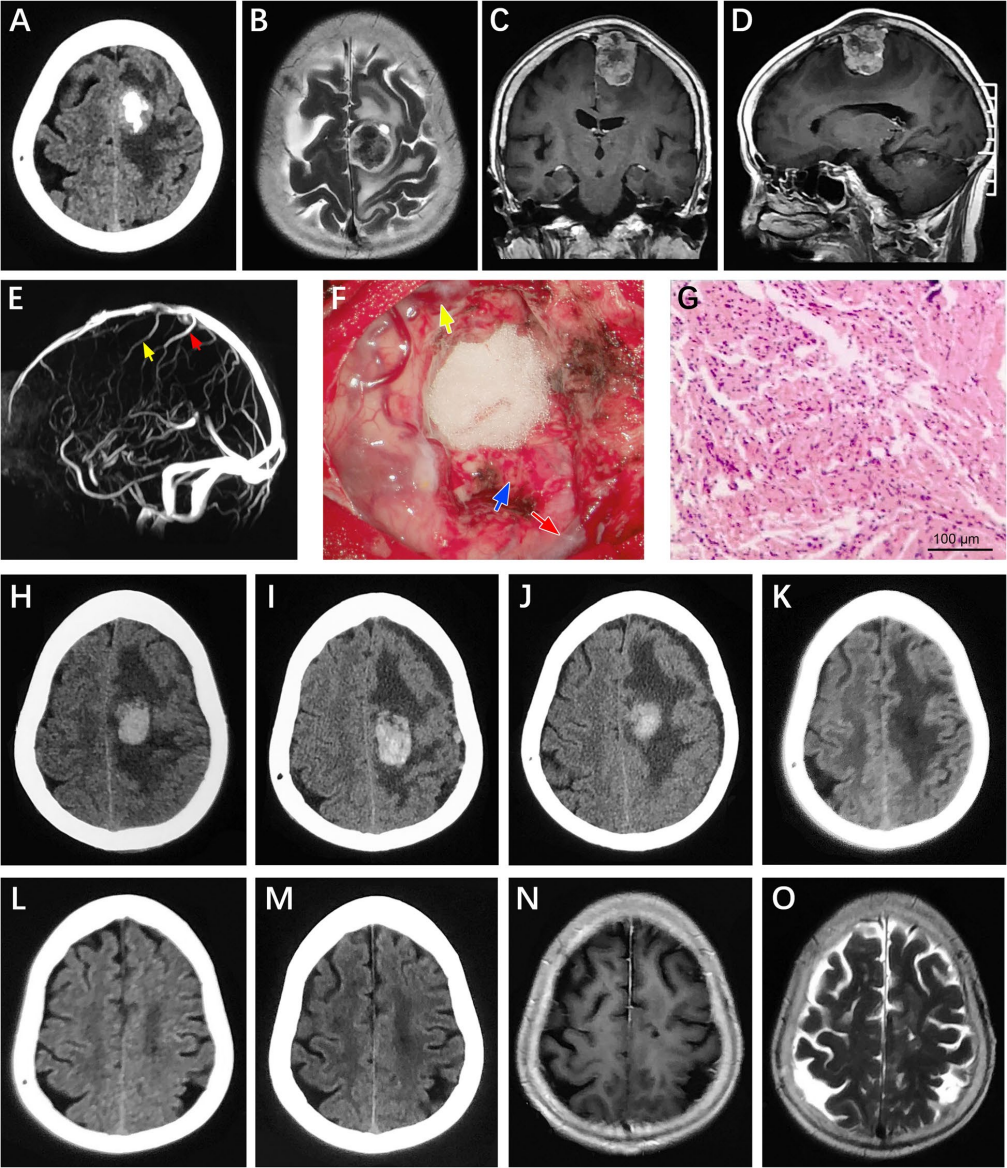
**A** CT showed intratumoral calcification. **B **Peritumoral edema was obvious. **C**-**D **The tumor invaded the skull. **E **MRV showed peritumoral vein accompanying (yellow and red arrows). **F** During the operation, the tumor was completely removed, peritumoral vein remained intact (yellow and red arrows), and the tumor invaded the pia mater and brain tissue (blue arrows). **G **Pathology showed atypical meningioma; **H** A small amount of hematoma in the operative area one day after operation, sober with right side muscle strength level 5. **I **Drowsiness with right side muscle strength level 3 three day after operation. **J** Lethargy with right side muscle strength level 1 five day after operation. **K** Drowsiness with right side muscle strength level 3 ten day after operation. **L** Sober with right side muscle strength level 4 fifteen day after operation. **M** Right side muscle strength level 4 one month after operation. **N**-**O** Right side muscle strength level 5 three month after operation

A case of left frontal parasagittal meningioma with risk factors (peritumoral edema, peritumoral vein, high-grade meningioma, key vein) secondary to venous infarction after injury of cortical pial vein system (Fig. 2).

## Discussion

At present, it is believed that the cause of surgery-related CVI is injury to the cortical vein and venous sinus or cortical pial venous system [5–7]. (1) Meningiomas often occur near the venous sinus and cortical drainage vein where arachnoid granules gather. During the operation, the vein around the meningioma or the related venous system is damaged, and there are no anastomotic veins in the injured area, which may lead to secondary CVI. (2) Meningiomas are usually associated with dural vessels and cortical drainage veins or with the pial arteriovenous system. During the operation, the interface between the tumor and the arachnoid is separated, and the arachnoid and pia mater are destroyed, which may damage the venous system of the pia mater cortex and eventually lead to CVI. These two kinds of injury mechanisms are mutual cause and effect and they aggravate the degree of venous infarction. Statistical analysis showed that tumor location (superficial), peritumoral edema (moderate to severe), peritumoral vein (key vein) and histological grade (WHO II–III) were risk factors for cerebral venous infarction, and the risk factors were closely related to the mechanism of the CVI.

### Tumor localization and peritumoral vein

Superficial meningioma refers to the convexity, parasagittal and falx, often accompanied by peritumoral veins. The growth pattern spreads along the cistern and embeds into the brain parenchyma, indicating that superficial meningioma has a wider contact surface between the tumor and brain parenchyma than deep meningioma and it lacks a cerebrospinal fluid barrier [8]. Therefore, the probability of injuring the peritumoral vein and cortical pial system during resection is higher than that for deep or other meningiomas.

The previous literature has reported that the bifrontal or near midline approach is a risk factor for cerebral venous infarction. The essence of the approach is to remove the tumor through the bridging vein and sinus, which hinders the exposure or manipulation of the tumor during the resection process so that the operator has to block or accidentally damage the vein around the tumor. At the same time, the previous view was that the frontal cortical vein and the anterior 1/3 superior sagittal sinus could be sacrificed, but clinical cases confirmed that this is definitely not desirable and the risk of infarction after injury will be significantly increased, which may have catastrophic consequences. Previous research [9] has shown predictors of positive motor function in rolandic meningomas, including venous involvement. To prevent CVI, the peritumoral veins and collateral veins of convexity, parasagittal and falx meningiomas can be classified [10–12]. It was suggested that the key veins should be avoided as much as possible, and the collateral veins should be selectively severed according to the degree of compensation to avoid the occurrence of cerebral venous infarction to the greatest extent.

### Peritumoral edema and histological grade

Peritumoral edema is a common imaging manifestation in patients with meningioma and is a compensatory reaction of damaged brain tissue. The pathogenesis of peritumoral edema includes brain parenchymal compression, secretion of fluids, venous compression and hydrodynamics theory. The presence of brain parenchyma tumor interface-associated edema indicates a poor prognosis of the nervous system [13]. The destruction of the tumor brain arachnoid layer interface leads to the formation of peritumoral edema. Dysplasia of the peritumoral drainage veins can also cause peritumoral edema. Meningiomas with peritumoral edema have a higher probability of destroying the cortical pial venous system. WHO grade II–III (atypical, anaplastic) meningiomas invade the arachnoid, pia mater, brain tissue and adjacent dura mater [14], increase the permeability of the meninges and blood vessels, aggravate the edema, and more easily damage the cortical venous system during the operation, resulting in increased postoperative edema and possibly venous infarction. Therefore, high-grade meningioma and peritumoral edema are risk factors for cerebral venous infarction. During the operation, we should try to separate along the tumor arachnoid boundary and retain part of the cortical venous system in order to reduce the incidence and severity of venous infarction.

### Tumor size and the extent of resection

Previously, it was reported that tumor growth could destroy the arachnoid interface between tumors and meninges, and tumors ≥ 4 cm are a risk factor for cerebral venous infarction [15], but our data showed that there was no significant difference. Considering that tumors increase in volume, the brain tissue is compressed, the blood–brain barrier is destroyed, and peritumoral edema is formed, but there are still some tumors without peritumoral edema. In addition, its formation may be related to the operation. Usually, for large tumors, we should reduce the tension of the tumor on the vein first, and the possibility of injury is small, which is not different from that of tumors < 4 cm. The data from this group confirmed that there was no difference in the degree of tumor resection. Usually, the residual tumor is closely related to the sinus. For sindou [16] I–III tumors, total resection can be achieved, while for sindou VI–V tumors, surgical resection is limited to reduce the risk. At the same time, the stenosis or occlusion of the sinus will be compensated for by collateral anastomosis. Therefore, the degree of resection is not a risk factor for CVI.

### Perioperative peritumoral vein protection

The following strategies can be selected during the perioperative period. MRV or CTV were performed routinely before operation, and a three-dimensional image fusion model (3D) [17] could be established if possible. Intraoperative neurophysiological monitoring and indocyanine green angiography (ICGVA) [18] were used to evaluate the spatial relationship between peritumoral veins and meningiomas, as well as the degree of tumor invasion and venous patency, so as to provide strong evidence for the protection and choice of peritumoral veins during operation. Venous anastomosis was performed after injury [19] when necessary.

### Pathophysiology and prognosis

Injury to a key vein or the cortical pial vein system can cause CVI, but the severity of CVI after injury differs, and the symptoms of patients are also different, from no obvious symptoms to serious neurological impairment and even disturbances of consciousness. Robertson [20] divided cerebral venous infarction into the acute phase and chronic phase. Severe complications occurred in a short time after the operation in the acute stage and were life-threatening, while the chronic stage lasted for several days to months with mild symptoms. Generally, injury to the critical vein can cause serious complications in the acute stage, including postinfarction hemorrhage, limb and speech dysfunction, epilepsy, coma, etc. Timely review of CT and surgical intervention are needed. After cortical pial venous system injury, the symptoms of the patients gradually worsened, but the symptoms could be relieved after conservative treatment. The prognosis of patients with venous infarction is better if they are treated in time, but damage to a key vein will cause irreversible neurological damage.

## Conclusion

Superficial meningioma, moderate to severe peritumoral edema, peritumoral critical vein and WHO grade II-III are independent risk factors for CVI. We should realize that postoperative CVI may cause serious complications. The key peritumoral veins and collateral veins should be protected as much as possible to avoid the occurrence of a postoperative CVI. Separation should be performed according to the tumor arachnoid interface to prevent injury to the cortical venous system. If an adhesion between the tumor and brain parenchyma is obvious, selective electrocoagulation can be used to reduce the probability of a CVI. In short, patients with high-risk factors need to be closely observed for any changes and receive timely intervention when necessary to ensure a good prognosis. 

## Data Availability

The datasets used and/or analyzed during the current study are available from the corresponding author on reasonable request.

## Abbreviations

CVI: Cerebral venous infarction
MRI: Magnetic resonance imaging
CT: Computed tomography
EI: Edema index
GOS: Glasgow outcome scale
GCS: Glasgow coma score
ICGV: Indocyanine green videoangiography
